# Long-term survival of *Babesia microti* and *Borrelia burgdorferi* in C3H/HeJ mice and their effect on Lyme arthritis and babesiosis manifestations

**DOI:** 10.1128/spectrum.00252-25

**Published:** 2025-08-12

**Authors:** Sandra C. Rocha, Mohamed A. M. Moustafa, Clara Vásquez Velásquez, Onyedikachi C. Azuama, Kashaf Zafar, Cory Meyer, Michael Araujo, Kyle Taylor, Nikhat Parveen

**Affiliations:** 1 Department of Microbiology, Biochemistry and Molecular Genetics, Rutgers New Jersey Medical School 12286, Newark, New Jersey, USA; 2 Veterinary Microbiology and Pathology, Washington State University College of Veterinary Medicine 70739, Pullman, Washington, USA; University of North Dakota 3579, Grand Forks, North Dakota, USA

**Keywords:** *Borrelia burgdorferi*, *Babesia microti*, co-infection, Lyme arthritis, spirochete, Apicomplexan protozoan

## Abstract

*Babesia microti* is increasingly co-transmitted with Lyme disease-causing *Borrelia burgdorferi* by ticks in the endemic regions of the United States. Infection in mice by this parasite mirrors human babesiosis, such as anemia, splenomegaly, alterations in splenic leukocyte balance, and diminished humoral immunity associated with enhanced Lyme disease manifestations at the acute phase. To evaluate the long-term survival of *B. burgdorferi* and *B. microti* in mice and their effects on pathogenesis, we conducted a 16-week infection experiment in C3H/HeJ mice. All mice infected with *B. microti,* irrespective of the co-infection, displayed a low-level, albeit microscopically detectable, parasitemia in both male and female mice even after 16 weeks post-infection. Splenomegaly was detected at this stage in both male and female mice and was significantly higher in females infected solely with *B. microti* compared to co-infected mice, likely due to a greater peak parasitemia at the acute phase of infection and persistent splenic manifestations in these mice. Interestingly, *B. microti* disrupted the blood-brain barrier (BBB) in mice, as reported during cerebral malaria caused by *Plasmodium falciparum*. Furthermore, *B. burgdorferi* colonization could be detected until 16 weeks of infection, while more pronounced Lyme inflammatory arthritis was observed at 4 weeks post-infection. This study underscores the complex interactions between *B. microti* and *B. burgdorferi* to affect each disease, highlighting the potential implications for vaccine development against Lyme spirochetes and therapeutic management of co-infected individuals.

**IMPORTANCE:**

Tick-borne co-infections are becoming increasingly prevalent worldwide due to simultaneous or sequential acquisition and transmission by *Ixodes* species ticks during their blood meal. We reported that *B. burgdorferi* and *B. microti* co-infection reciprocally affects these pathogens during the acute phase of infection; however, the effect of co-infections on microbial long-term persistence in the murine model was not previously investigated. In this study, we have filled a critical lacuna in understanding the interactions between these two pathogens at different stages of infection and their effects on the host and disease manifestations in mice. Our investigation provides insights into their pathogenicity to allow the development of effective vaccines and successful antimicrobials against these tick-borne co-infections.

## INTRODUCTION

Lyme disease (LD), caused by the extracellular spirochetal bacterium *Borrelia burgdorferi*, is the most prevalent tick-borne disease in the United States, primarily in the Northeastern and upper Midwestern regions, with an estimated number of 476,000 Lyme disease cases reported by CDC per year (1). It is a systemic disease in which spirochetes can spread and persist in different organs and tissues and stimulate host inflammatory immune responses to develop cardiovascular, arthritic, neurologic, and dermatologic manifestations. The most common symptom of long-term LD in the United States is arthritis, which affects large joints, particularly the knees (2, 3). *Babesia* species are intraerythrocytic Apicomplexan protozoan parasites that are also prevalent in tick-infested regions of the USA, with 2,418 cases reported in 2019. Most cases in North America are caused by *Babesia microti*. In 2020, there was a 24% decrease in reported babesiosis cases, likely influenced by the COVID-19 pandemic, which potentially decreased *Babesia* diagnosis and its transmission rates (4). Infections caused by *Babesia* species vary in severity, ranging from asymptomatic infection in immunocompetent individuals to severe disease manifested in the form of hemolytic anemia, splenomegaly, and thrombocytopenia, especially in immunocompromised and elderly individuals. In fact, severe babesiosis is often associated with immunodeficiency caused by splenectomy, malignancy, and immunosuppressive therapy such as during organ transplant and results in high morbidity and even mortality (5). Reports of spontaneous splenic rupture in undiagnosed people, who were otherwise healthy, indicate the severity of outcomes of *Babesia* infection or co-infection on hosts (6–12).

Selection of the LD treatment regimen is based on the patient’s prognosis. In recently published guidelines, the most important disease manifestations were considered, including erythema migrans, neurologic LD, Lyme carditis, and Lyme arthritis for LD treatment recommendations (13). For the initial treatment of Lyme arthritis, oral antibiotic therapy (such as doxycycline, amoxicillin, or cefuroxime) for 28 days is strongly recommended; however, some patients are reported to develop antibiotic-refractory Lyme arthritis despite combined oral and intravenous treatments. In such cases, disease-modifying antirheumatic drugs (DMARDs), intra-articular steroids, or arthroscopic synovectomy could also be employed for more severe cases (13, 14). The preferred treatment regimen for babesiosis involves the combination of atovaquone and azithromycin, which must last 7 to 10 days but can be extended for at least 6 consecutive weeks for highly immunocompromised patients. Treatment is considered complete when *Babesia* is absent in blood smears for 2 consecutive weeks (15). Co-infections involving *B. burgdorferi* and *B. microti* should also be considered when LD patients exhibit characteristic laboratory abnormalities, such as thrombocytopenia, leukopenia, neutropenia, and/or anemia, along with persistent high-grade fever (13).

*B. burgdorferi* and *B. microti* can coexist in ticks and reservoir host(s) to facilitate co-transmission to humans mostly by *Ixodes scapularis* ticks in North America (16). In addition, *B. burgdorferi* can promote the transmission of *B. microti,* increasing the prevalence of babesiosis in regions that are endemic for LD (17). Serological studies from New York and New England reported that among individuals with acute LD, 2% to 19% had co-infection with *B. microti* (18–20). An even higher co-infection rate was detected in a two-tiered serological study conducted in New York in 2016, where 28.6% of LD-positive individuals also showed antibody reactivity to *B. microti* (21). Although limited data are available from endemic states about co-infections, serological tests do not always detect active infection. For example, in New Jersey, multiplex qPCR assays conducted on samples collected from three LD clinics detected the presence of both *B. burgdorferi* and *B. microti* simultaneously. Together with clinical data, these results indicated active Lyme disease (LD) and babesiosis in a significant number of patients. In that study, we showed an active co-infection rate of 38.5% (22). However, the CDC only recognizes metagenomic PCR and sequencing for the diagnosis of active Lyme disease (23). An increase in both severity and duration of illness was reported during co-infections when compared to individuals infected only with *B. burgdorferi* or *Babesia* (17, 19).

Co-infection studies in murine models have significantly enhanced our understanding of the reciprocal effects of *B. burgdorferi* and *B. microti* on disease pathogenesis (24–27) despite limited data availability for humans. C3H/HeJ mice have mutations in the TLR4 gene, resulting in defective LPS signaling (28) compared to C3H/HeN mice, which possess fully functional TLR4. *B. burgdorferi* is known to have an atypical form of LPS or, more accurately, a glycolipid with structural differences from classical LPS found in gram-negative bacteria. However, the presence of true LPS in *B. burgdorferi* remains debatable, while the abundance of lipoproteins in this spirochete plays a more prominent role in immune modulation (29). Thus, the presence of atypical LPS confers resistance to this endotoxin (28), differing from mouse susceptibility to gram-negative bacterial infections (30–32). As early as 1993, infection of C3H/HeJ mice with the N40 strain showed higher inflammatory arthritis than C3H/HeN after 180 days (33). We confirmed these results and found that *B. microt*i infection did not appear to signal through TLR2 or TLR4 and exhibited similar levels of anemia, splenomegaly, and parasitemia in both strains of mice (26). Unlike *Plasmodium* species, glycosylphosphatidylinositol (GPI) anchor on the merozoite surface of *B. microti* appears to signal through either TLR2 or TLR4 (34–37). Experimentally co-infected C3H mice showed a significant increase in spirochete burden and persistence in different organs at the acute phase, as well as an exacerbation of LD-associated inflammatory arthritis likely due to suppression of adaptive immune responses observed by *B. microti* (25–27). Interestingly, *B. microti* parasitemia was attenuated in co-infected C3H mice with TLR2-mediated innate immune response stimulated by *B. burgdorferi* lipoproteins, which appear to promote *B. microti* clearance in co-infected mice (25, 26).

The effects of *B. burgdorferi* and *B. microti* co-infection in murine models and their impact on pathogen presence in different tissues over short- and long-term durations have not been examined yet. We investigated the effect of co-infection using our bioluminescent *B. burgdorferi* N40D10/E9 derivative (N40 henceforth) (38) and *B. microti* gray strain (ATCC 30221) in both sexes of C3H/HeJ mice in this study to assess pathogenesis from the acute phase of infection (2 weeks) to long-term infection (16 weeks).

## MATERIALS AND METHODS

### *B. burgdorferi* and *B. microti* cultivation and inoculum preparation

The bioluminescent *B. burgdorferi* N40 strain was grown in BSKII medium containing 6% rabbit serum at 33^∘^C. When the culture reached the logarithmic phase, the N40 count was adjusted to 10^4^ spirochetes/mL and used 100 µL for injection (10^3^ spirochetes per mouse). The protozoan *B. microti* cannot be grown continuously *in vitro*. Therefore, its frozen stock was thawed and injected in C3H/SCID female mice to allow parasitemia to develop in blood. When parasitemia reached >20%, heparinized blood from the mouse was collected and diluted in PBS to obtain 10^5^ infected RBCs (iRBCs)/mL, and 100 µL of diluted blood (10^4^ parasites per mouse) was inoculated.

### Experimental design for co-infection assays in mice

**Fig 1:**
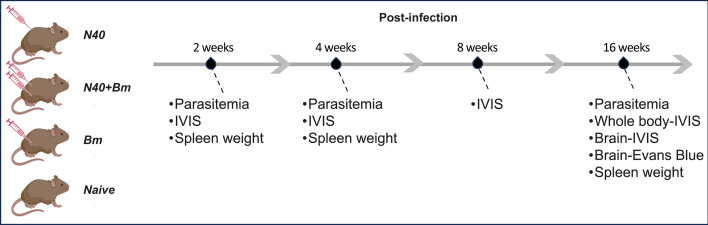
Co-infection experiment plan in C3H/HeJ mice and euthanasia time points. We carried out an infection experiment using 5-week-old C3H/HeJ mice for 4 months to determine if *B. burgdorferi* or *B. microti* (*Bm*) persists long term in co-infected rodent hosts and to ascertain their reciprocal effects on the disease’s pathogenesis at short- and long-term infection. C3H/HeJ female and/or male mice were injected with 100 µL containing 10^4^ *Bm-*iRBC subcutaneously (sc) in the left flank and/or infected with 10^3^ N40 spirochetes sc in the right flank and organized into four experimental groups: (i) N40; (ii) N40 + Bm co-infection; (iii) Bm; (iv) Naïve. Parasitemia determination by microscopy and IVIS were used at different time points to determine *B. microti* and N40 burdens, respectively. This experiment was conducted three times, first in both sexes of mice and then repeated two more times only in female mice to confirm the results.

We infected 5-week-old C3H/HeJ mice, as described above, to compare the effects of *B. burgdorferi-B. microti* co-infection on each pathogen and host at the acute phase (2 weeks), after generation of adaptive immunity (4 weeks), and during persistent infection (16 weeks). C3H/HeJ mice were purchased from Jackson Laboratory. We injected 100 µL of diluted heparinized blood recovered from SCID mouse in each animal containing 10^4^ iRBC subcutaneously (sc) in the dorsal flank of the left hind leg and with 10^3^ N40 spirochetes sc in the right flank, as described previously (25, 39). The mice were organized into four experimental groups: (i) Naive, (ii) N40 infection only, (iii) N40 + *B. microti* co-infection, and (iv) *B. microti* infection only (Fig. 1). Blood smears were prepared from mice infected with *B. microti* at different time points, and parasitemia was determined after staining with Giemsa stain by examining 25 microscopic fields (40). Colonization by N40 was monitored at different time points of infection by live imaging using IVIS-200 (Perkin Elmer, Waltham MA) after intraperitoneal injection of 200 µL of 30 mg/mL D-luciferin substrate in PBS, as previously described (38). Animals under anesthesia were bled by cardiac puncture for plasma collection and then euthanized at 2, 4, 8, or 16 weeks post-infection (pi). Various organs were aseptically harvested for further analysis (Fig. 1). The number of uninfected/infected mice euthanized at each time point is as follows: at week 2: 5 Naïve; 15 N40; 15 N40 + *B. microti*; and 15 *B. microti*, at week 4: 5 Naïve; 10 N40; 10 N40 + *B. microti*; 5 *B. microti*, at week 8: 5 Naïve; 5 N40; 5 N40 *+ B. microti*; 5 *B. microti*, and at week 16: 5 Naïve; 20 N40; 20 N40 *+ B. microti*; and 20 *B. microti*. Duplex quantitative polymerase chain reaction (qPCR) was conducted as described previously (26, 27). This experiment was conducted three times: first in both sexes, followed by two additional repetitions in female mice to confirm results and ensure reproducibility.

### Evaluation of blood–brain barrier permeability integrity

To evaluate the integrity of the BBB in mice infected with N40, *B. microti,* or N40 *+ B. microti,* mice under anesthesia were injected intravenously (iv) with 200 µL of 2% Evans Blue solution (diluted in PBS, pH 7.2) in the ventral tail vein. Mice were then perfused with 40 mL of PBS and then euthanized ~1 hour after Evans Blue injection and then imaged. Brains were then collected and immersed in a formamide solution for 48 hours at 37°C. The Evans Blue dye released in the formamide solution was quantified in 96-well plates by absorbance measurement at 620 nm. A standard curve using different concentrations of Evans Blue solution was prepared to calculate the amount of dye per milliliter of formamide, as previously described (41).

### Histopathology analysis

Joints were fixed using formalin solution, processed using 10% EDTA‐decalcification protocol, and 5 µm thick sections were then cut using a microtome. Sections were mounted on slides for hematoxylin and eosin (H&E) staining. Histopathological examination of the stained tibiotarsus joint sections was then conducted to assess inflammation.

### Statistical analysis

Data were processed using GraphPad Software version 9.5.1, and the significant differences between two groups were determined using two-tailed unpaired Student *t*-tests for unequal variances or one-way ANOVA, followed by Tukey’s *post hoc* test. Differences with *P* < 0.05 were considered statistically significant at a 95% CI.

## RESULTS

### *B. microti* parasitemia is attenuated at the early stage of co-infection, but a few parasites persist in blood long term

**Fig 2:**
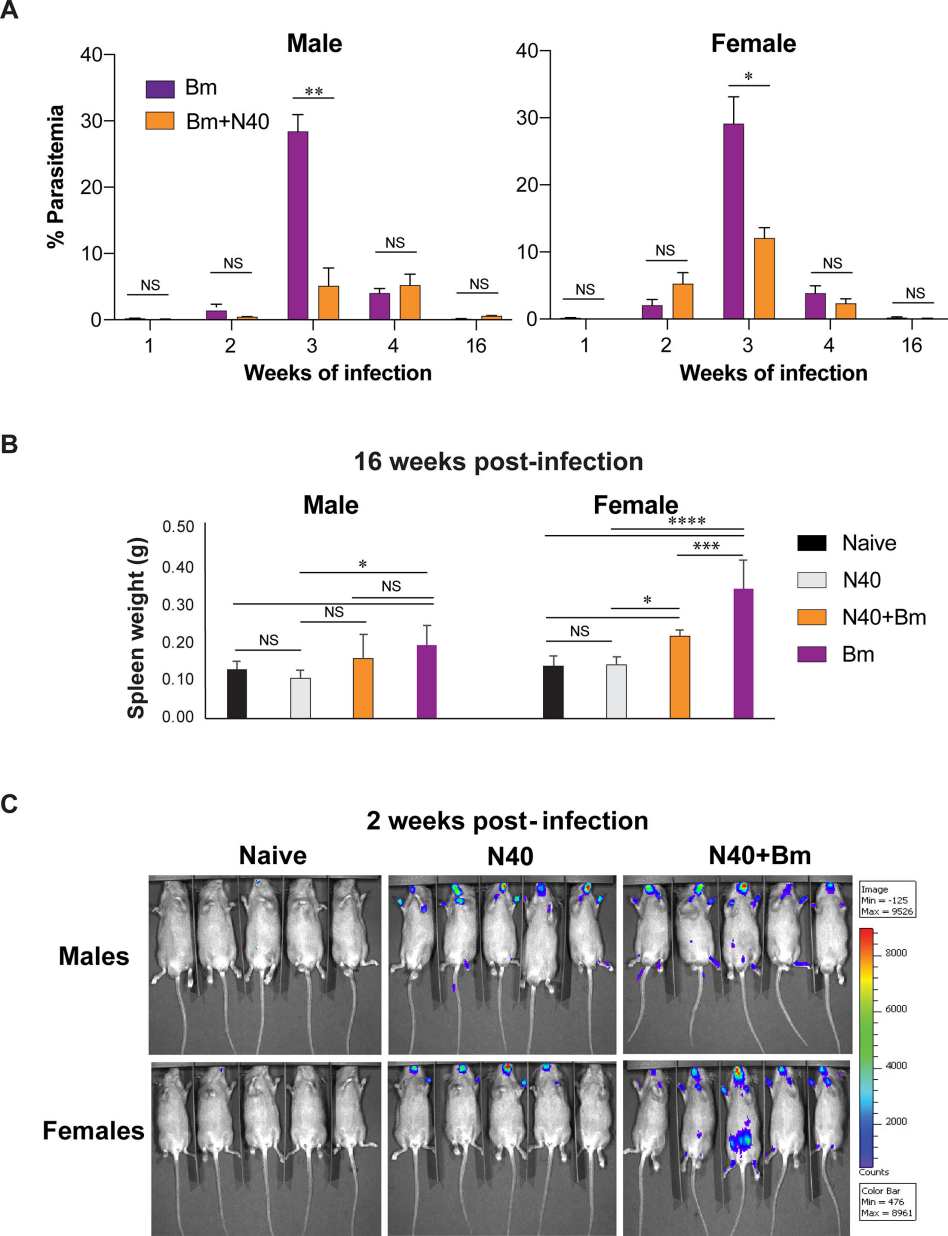
Comparative analysis of *B. microti* parasitemia and splenomegaly and *B. burgdorferi* colonization in male and female C3H/HeJ mice. (**A**) C3H/HeJ female and male mice were inoculated sc in the left flank with 10^4^ *Bm-*infected red blood cells (iRBCs) alone or together with 10^3^ N40 spirochetes (inoculated sc in the right flank). *B. microti* parasitemia was determined in blood smears stained with Giemsa stain and by calculation of the average percentage of iRBC of total RBCs observed in 25 microscopic fields using oil immersion at 1,000×. (**B**) After terminal bleeding, mice were euthanized by CO_2_ asphyxiation at 16 weeks post-infection. Spleens were aseptically harvested and weighed to determine splenomegaly. (**C**) The effect of *Bm* infection on N40 colonization was determined in both sexes of mice infected with N40 and/or *Bm* using IVIS-200 after intraperitoneal injection of 200 µL of 30 mg/mL D-luciferin at 2 weeks post-infection. The presence of *B. burgdorferi* N40 strain was visualized in different tissues measured by bioluminescent radiance as a semi-quantitative indicator of colonization by spirochetes. Parasitemia and splenomegaly data are represented by mean ± s.e.m. and were processed using GraphPad Software version 9.5.1. Significant differences between the two groups were tested by using two-tailed unpaired Student *t*-tests for unequal variances for parasitemia data and one-way ANOVA, followed by Tukey’s *post hoc* for analysis to evaluate splenomegaly. Statistical differences with *P* < 0.05 were considered statistically significant at 95% confidence interval (NS, not significant, *P < 0.05, **P < 0.01, ***P < 0.001, and ****P < 0.0001).

Our experimental plan and sample analyses conducted are outlined in Fig. 1. We monitored the parasitemia in male and female mice up to 16 weeks (Fig. 2A). The peak of *B. microti* parasitemia occurred at ~3 weeks pi and had comparable levels between male and female mice. Consistent with previous reports, lower-peak parasitemia was observed when mice were co-infected with the *B. burgdorferi* N40 strain in both males and females compared to those infected solely with *B. microti*. This difference was most pronounced in males at this stage, with co-infected mice (N40 + *B. microti*) showing 5.1% ± 2.7% compared to 28.4% ± 2.6 % parasitemia in mice infected with *B. microti* alone (Table 1). Co-infected female mice showed 12.0% ± 1.6% parasitemia compared to 29.1% ± 4% in *B. microti* singly infected mice. Thus, the difference in parasitemia levels in *B. microti* infected and co-infected mice in both sexes was statistically significant at this stage of infection (Fig. 2A and Table 1). Less than 1% iRBCs were still detected microscopically at 16 weeks pi in all *B. microti* infected/co-infected groups, with differences among them not observed to be statistically significant (Table 1, Fig. 2A).

**TABLE 1:** *B. microti* parasitemia at different stages of infection/co-infection with the *B. burgdorferi* N40 strain^*a*^

Time point (weeks)	*Bm* parasitemia (% ± SEM)					
	Male			Female		
	*Bm*	N40 + *Bm*	*P*-value	*Bm*	N40 + *Bm*	*P*-value
1	0.2 ± 0.06	0.1 ± 0.06	0.288	0.2 ± 0.03	0.1 ± 0.0	0.184
2	1.9 ± 0.41	0.4 ± 0.09	0.061	2.0 ± 0.90	5.2 ± 1.70	0.193
3	28.4 ± 2.55	5.1 ± 2.71	0.003	29.1 ± 3.98	12.0 ± 1.59	0.036
4	4.0 ± 0.71	5.2 ± 1.69	0.564	3.8 ± 1.13	2.3 ± 0.71	0.325
16	0.1 ± 0.03	0.6 ± 0.12	0.060	0.2 ± 0.10	0.1 ± 0.03	0.313

^*a*^ Each value represents the average ± the standard error of the mean (SEM).

### Babesiosis-associated splenomegaly is lower in male mice at 16 weeks of infection

We have previously shown that *B. microti* infection affects the splenic architecture in mice, causing splenomegaly (39), which was also observed in humans (42) and that N40 + *B. microti* co-infected female C3H/HeJ mice had pronounced splenomegaly at 3 weeks of infection and was comparable to *B. microti*-infected mice (27). However, it is still not clear if the splenomegaly is sustained during long-term co-infection in male and female mice (16 weeks). Although both male and female mice infected solely with *B. microti* showed an increase in splenic mass compared to the N40-infected group at 16 weeks pi (Fig. 2B), splenomegaly was significantly higher only in *B. microti* infected and co-infected female mice. Therefore, we conducted all follow-up experiments only in female mice to determine changes in pathogens levels and resulting disease manifestations.

**Fig 3:**
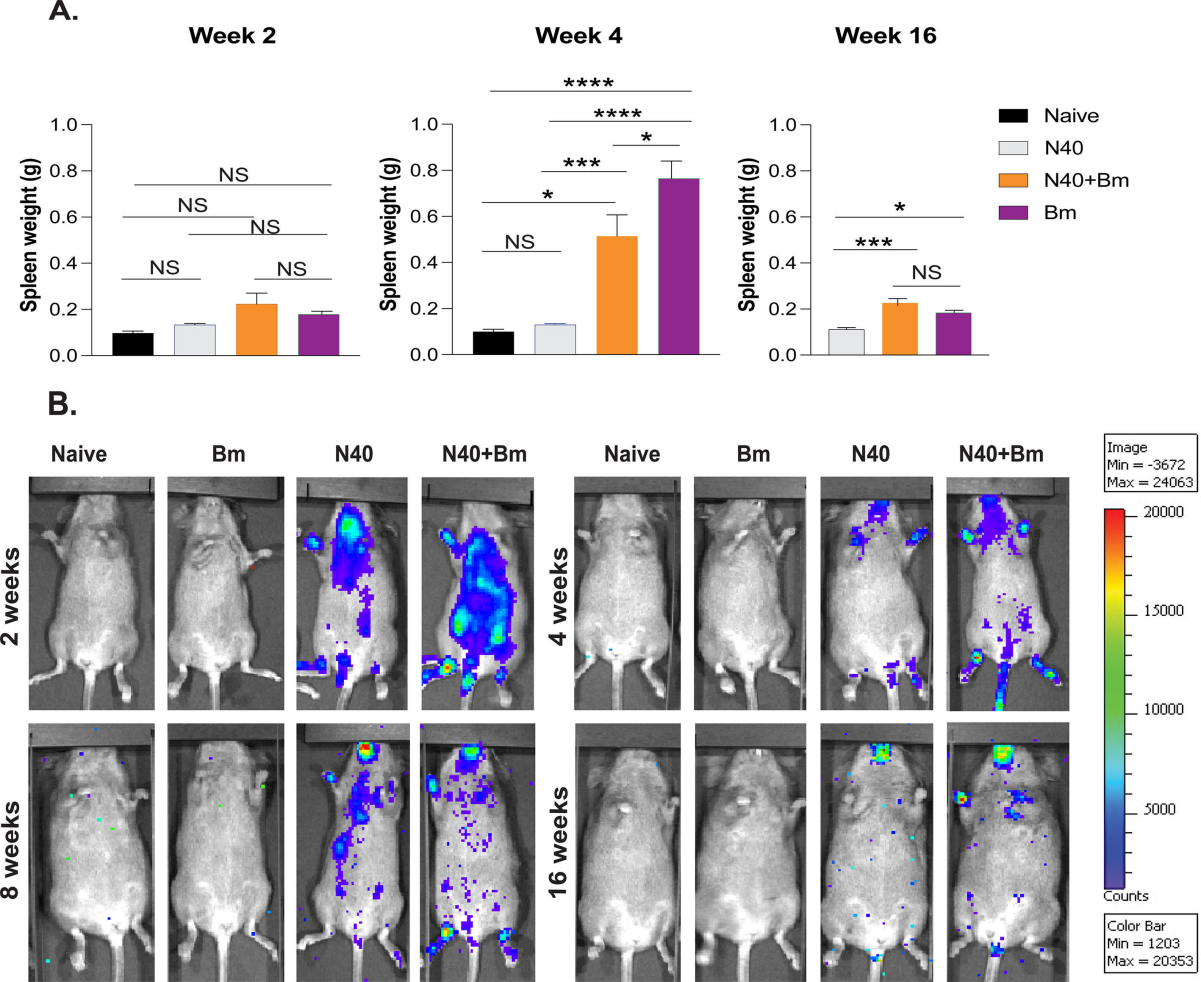
Babesiosis-associated splenomegaly and colonization by *B. burgdorferi* detected by live imaging at different stages of infection in C3H/HeJ female mice. (**A**) C3H/HeJ female mice were euthanized by CO_2_ asphyxiation at 2, 4, or 16 weeks of infection. Spleens were aseptically harvested and weighed for the determination of splenomegaly. The data represented by mean ± s.e.m. of spleen weights were processed using GraphPad Software version 9.5.1. Significant differences between the two groups were tested by using one-way ANOVA, followed by Tukey’s *post hoc* test. Statistical differences with *P* < 0.05 were considered significant at 95% CI (NS, not significant, *P < 0.05, **P < 0.01, ***P < 0.001, and ****P < 0.0001). (**B**) To determine the effect of the *Bm* infection on N40 colonization, live imaging of C3H/HeJ female mice groups infected with N40 and/or *B. microti* was determined using IVIS-200 (PerkinElmer) after intraperitoneal (ip) injection of 200 µL of 30 mg/mL D-luciferin at 2, 4, 8, or 16 weeks pi. Based on the net radiance photon measurement, one mouse was selected from each group to represent the average bioluminescence.

Females were then examined closely at different time points of infection. Although infected females had a slight increase in spleen weights during early infection (2 weeks pi) in *B. microti-*infected and co-infected mice, differences among groups were not statistically significant (Fig. 3A). At 4 weeks pi, relative to naïve (0.1 ± 0.01 g) and N40-infected mice (0.13 ± 0.005 g), *B. microti* significantly increased splenomegaly in co-infected mice to 0.51 ± 0.09 g (a 5.1-fold increase relative to naïve mice, *P* = 0.014), but it remained significantly lower than in *B. microti*-infected mice, which exhibited the most severe splenomegaly, with an average spleen weight of 0.77 ± 0.07 g (a 7.7-fold increase relative to naïve mice, *P* < 0.0001). By 16 weeks pi, splenomegaly appeared to be largely resolved in male mice, with minimal to no differences in spleen weight between co-infected and *B. microti*-infected mice at this time point while differences were observed in splenomegaly in females in different experiments (Fig. 2B and 3A). Thus, *B. burgdorferi* infection can begin to attenuate splenomegaly in co-infected mice at 4 weeks pi, with its effect diminishing long term in both sexes.

### Detection of live *B. burgdorferi* presence in mice at different stages of infection

Our laboratory has generated stably bioluminescent *B. burgdorferi* N40 strain. This strain (38) allowed us to detect colonization by *B. burgdorferi* at different stages of infection using the same set of mice. Two weeks pi, N40 colonization appeared in similar organs of both male and female mice, albeit some variation was observed in different mice (Fig. 2C), likely influenced by relatively higher *B. microti* parasitemia in these mice. Colonization levels of *B. burgdorferi* in this experiment were comparable in both sexes of mice.

**Fig 4:**
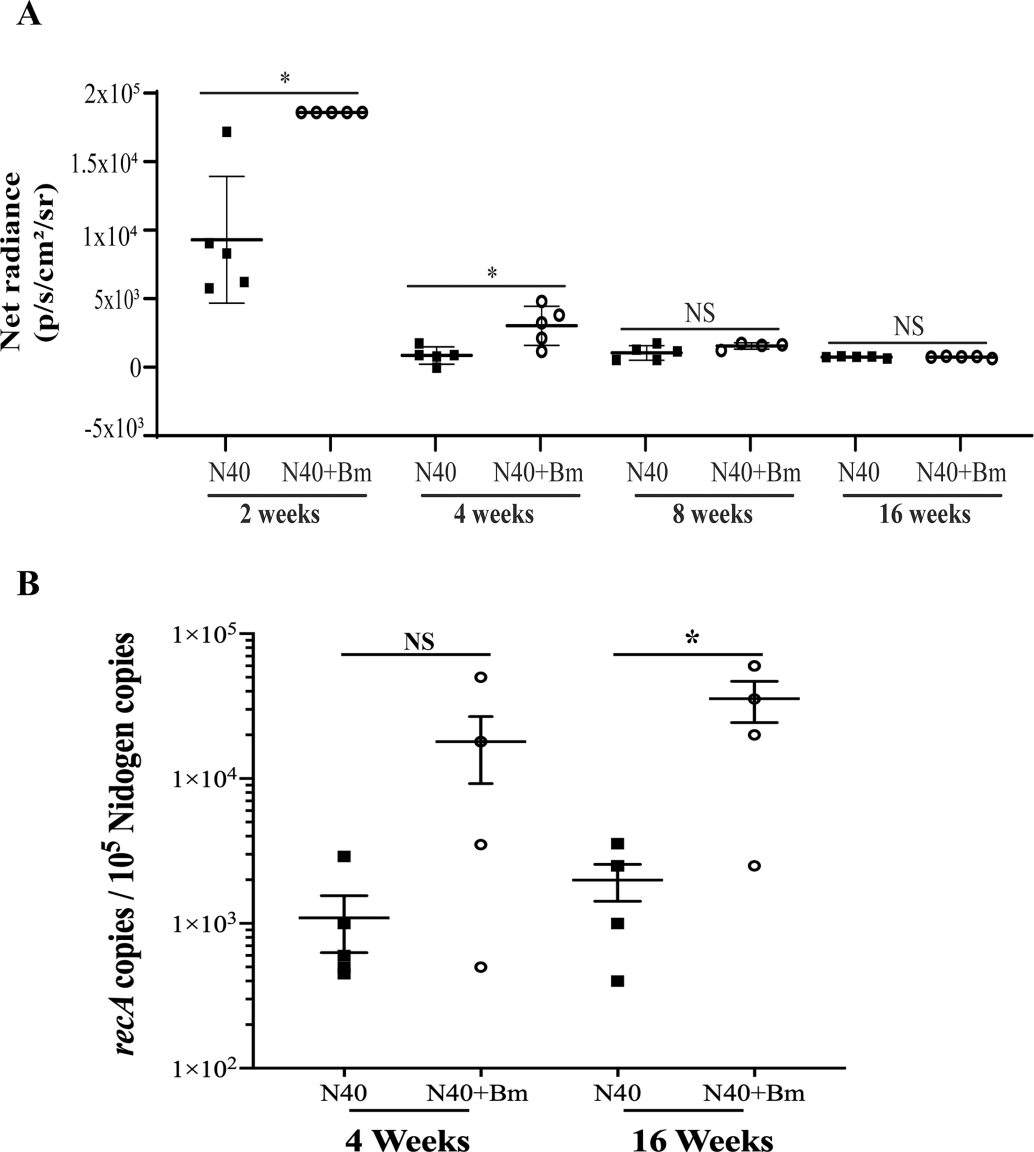
Effect of *B. microti* co-infection on *B. burgdorferi* N40 colonization levels in female C3H/HeJ mice. (**A**) Live imaging of C3H/HeJ female mice infected with *B. burgdorferi* N40 and/or *B. microti* was conducted to detect bioluminescence. Quantification of radiance associated with stably bioluminescent N40 colonization is presented by measuring net radiance (photons/s/cm²/sr) in five mice from each group obtained after deducting values from naïve mice. (**B**) Burden of *B. burgdorferi* was determined in joints of infected mice by employing duplex qPCR using *recA* gene of spirochetes and nidogen gene of mice for quantification using specific molecular beacon probes for each gene. In both panels **A** and **B**, data were averaged, and standard errors determined the range of radiance obtained at different time points. Significant differences between the two groups were tested by using two-tailed unpaired Student *t*-tests for unequal variances. Statistical differences were considered statistically significant at 95% CI and are marked by asterisks (NS, not significant, *P < 0.05).

Because we did not observe noticeable sex-based differences in N40 colonization, we repeated further studies only in female mice. We captured live images from N40-infected and N40 *+ B. microti* co-infected mice at 2, 4, 8, and 16 weeks pi (Fig. 3B). In this experiment, spirochetes were detected in whole mice, with the bioluminescent signal intensity levels consistently higher in the co-infected mice at 2 weeks compared to the N40-infected group. These differences diminished significantly or were eliminated during later stages of infection (4, 8, and 16 weeks pi) when both groups showed detectable albeit comparable levels of N40 colonization. Naïve mice showed no bioluminescence (Fig. 3B), as expected, and served as the background to determine net radiance calculations (Fig. 4A and Fig. S1). *B. microti*-infected mice were also included for imaging (Fig. 3B). Thus, *B. microti* infection increases the burden of *B. burgdorferi* at the acute phase (2 weeks) of infection; however, this effect of *B. microti* presence normalizes and does not persist during long-term co-infection.

To further quantify *B. burgdorferi* DNA copies using qPCR, we used *recA* gene copy numbers normalized to 10^5^ copies of mice nidogen gene (Fig. 4B). The pattern observed with bioluminescence measurements (Fig. 4A) was largely reflected by DNA copy determination; however, some differences were noticed due to higher animal-to-animal variation in co-infected groups. We cannot rule out the possibility of detecting dead *B. burgdorferi* DNA remaining in joints by qPCR, which contributes to the slight differences observed between these two assays.

### *B. microti* infection compromises the BBB integrity like related parasites

**Fig 5:**
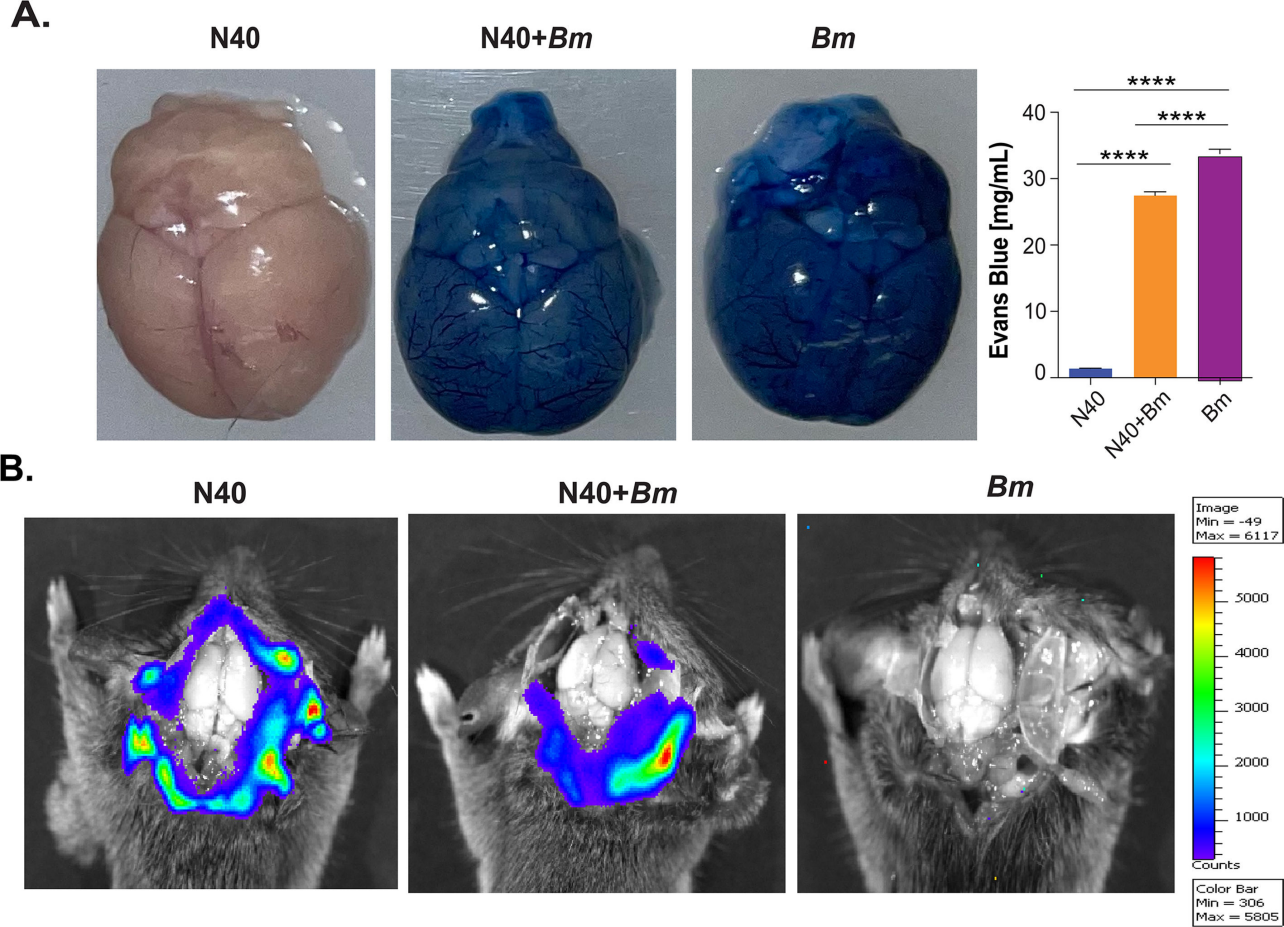
*B. microti* infection disrupts BBB integrity and *B. burgdorferi* colonization in dura mater persists irrespective of co-infection with *B. microti*. (**A**) To evaluate the integrity of the BBB, penetration of Evans blue dye was evaluated at 2 weeks pi. The BBB disruption is indicated by blue color remaining in the brain even after perfusion with PBS. The brains were then immersed in formamide solution for 48 hours at 37°C to release Evans blue dye, which was quantified in 96-well plates by optical density measurement at 620 nm with a spectrophotometer. A standard curve for Evans Blue with known values was determined to calculate the weight of dye (mg)/formamide (mL). The graph bars at the right side of panel A represent mean ± SD of the dye (mg/mL) released in the formamide solution. Significant differences between the two groups were determined by one-way ANOVA, followed by Tukey’s *post hoc* test. Statistical differences with *P* < 0.05 were considered statistically significant at 95% CI (NS, not significant, ****P < 0.0001). (**B**) At 2 weeks post-infection, bioluminescent images of brains from C3H/HeJ female mice infected with N40 alone or together with *B. microti* were determined using IVIS-200. Mice were anesthetized with isoflurane and intracardially injected with 200 µL 30 mg/mL D-luciferin substrate. After 5 minutes, the animals were euthanized, and skulls opened for acquisition of images for bioluminescence detection in dura mater and brain.

Evans Blue dye cannot cross the BBB unless its integrity is compromised. In this context, blue stain deposited in the mouse brain suggests BBB disruption, as has also been observed for *Plasmodium berghei* infection in mice (41). Here, we assessed the integrity of mice BBB at acute phase (at 2 weeks pi) in female mice and found that the brain from N40-infected mice demonstrated no Evans blue dye penetration (1.43 ± 0.03 mg/mL), indicating that *B. burgdorferi* infection does not compromise the BBB integrity (Fig. 5A). N40 *+ B. microti* co-infected mouse had blue dye accumulation throughout the brain (left), with high recovery of dye in formamide (27.47 ± 0.55 mg/mL, *P* < 0.0001 compared to N40) and also from *B. microti*-infected mouse (33.77 ± 0.66 mg/mL*, P* < 0.0001 compared to N40). Dye accumulation was significantly higher (*P* < 0.0001) in *B. microti* infected compared to the co-infected mice, even though parasitemia was comparable in these groups of mice. We also captured bioluminescent images from female mice brains at the early stage of infection (2 weeks pi) and observed *B. burgdorferi* colonizes dura mater in both N40-infected and N40 *+ B. microti-*infected mice (Fig. 5B) in a comparable manner (total 12,206.8 p/s/cm²/sr for N40 infection and 10,566.8 p/s/cm²/sr for co-infection). These findings suggest that, like *Plasmodium* spp., *B. microti* also compromises the integrity of the BBB, potentially allowing other pathogens to enter the brain.

### Lyme inflammatory arthritis is more pronounced at 4 weeks compared to 16 weeks of infection

**Fig 6:**
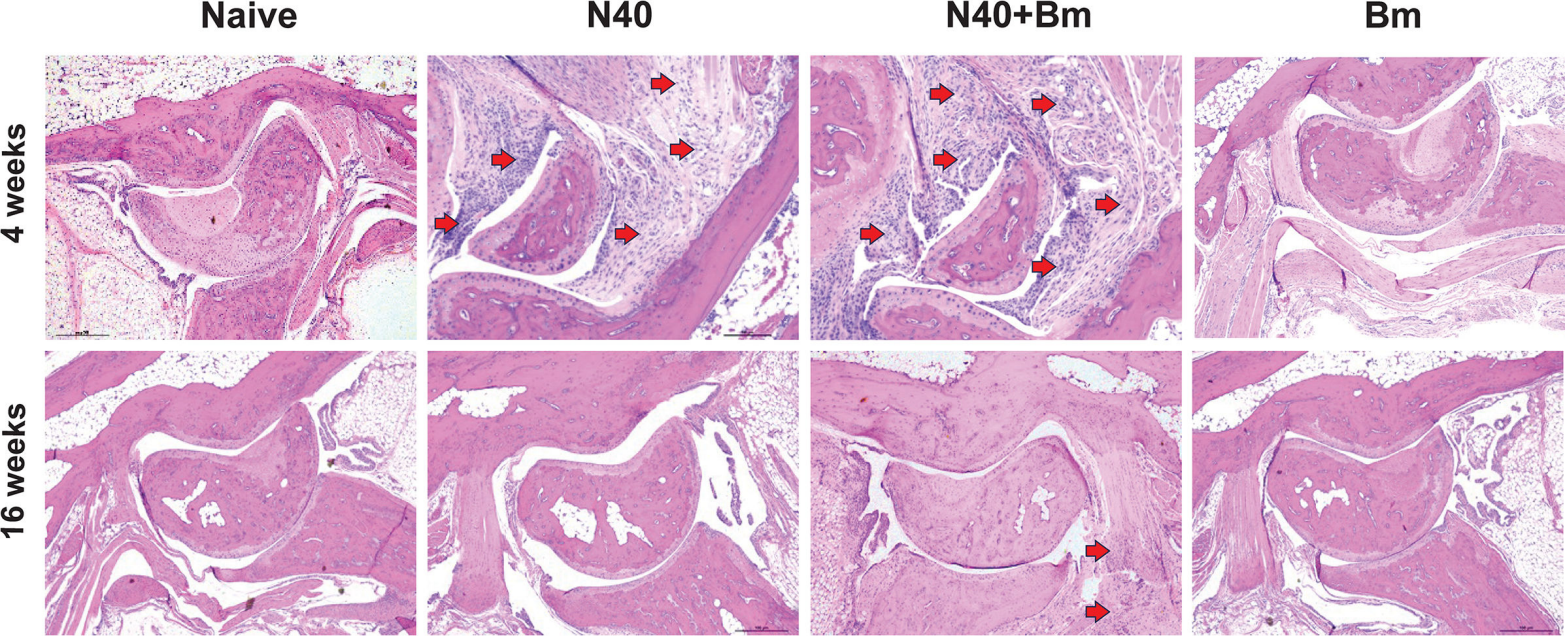
*B. microti* co-infection enhances inflammatory Lyme arthritis at the early stages of infection**.** Joints from Naïve or C3H/HeJ female mice infected with *B. microti*, N40 alone or together with *B. microti* were harvested at 4 and 16 weeks of infection and prepared for histopathological analysis. Joints were first fixed with formalin, processed using the 10% EDTA-decalcification protocol, and 5-μm-thick sections were cut by a microtome and then mounted on slides for hematoxylin and eosin (H&E) staining. The joints from co-infected mice had higher leukocyte infiltration (indicated by red arrows) compared to N40 singly infected mice, which was more apparent at 4 weeks pi; however, overall histopathology scoring rates were comparable. Inflammation almost completely resolved in all N40-infected and co-infected mice at 16 weeks pi (Table 2).

Lyme inflammatory arthritis is a common manifestation observed in individuals with long-term LD in the United States (2) and in experimentally infected C3H/HeJ mice. In addition, more severe inflammation of joints was observed in mice co-infected with *B. microti* at the early phase of infection (27). In our experiments here, histopathological examination of the tibiotarsus joint sections at 4 weeks pi showed significant leukocyte infiltration in the joints in N40-infected and N40 + *B. microti* co-infected mice. Overall, histopathological scores for inflammatory Lyme arthritis were found to be comparable in both N40-infected and co-infected mice at 4 weeks, and the difference between these two sets of mice was not observed even at 16 weeks pi (Table 2); however, inflammation in joints was more pronounced at 4 weeks. During long-term infection (16 weeks pi) in two different experiments, both groups of mice showed resolution of inflammatory arthritis, reflecting reduced colonization levels by spirochetes observed at this late stage of infection/co-infection of mice. As expected, uninfected or *B. microti*-infected mice did not display inflammation in joints either at 4 or 16 weeks pi (Fig. 6 and data not shown).

**TABLE 2:** Histopathological scoring for inflammatory Lyme arthritis in the tibiotarsus of infected mice^*a*^

Experiment no.	Inoculum	Time point	Mouse number				
			1	2	3	4	5
1	N40	16 weeks	+	+	+	+	+
	N40 + Bm	16 weeks	+	++	+	+	+
2	N40	4 weeks	+++	+++	++	+++	+++
	N40 + Bm	4 weeks	+++	+++	+++	+++	++
	N40	16 weeks	++	+	++	+	
	N40 + Bm	16 weeks	++	++	+	++	

^*a*^ Tibiotarsus inflammation in *B. burgdorferi*-infected (N40) or co-infected (N40 + Bm) mice was evaluated on a scale from negative (−) to severe (+++), based on the following 4 criteria: (i) synovial hyperplasia; (ii) cartilage erosion; (iii) increased lymphocytic infiltration; (iv) alterations in synovial space, compared to naïve mice.

## DISCUSSION

Studies of the pathogenesis and host susceptibility are of great importance for the development of promising vaccines and antimicrobial compounds against tick-borne diseases, especially during co-infections. There are several factors related to the host genetic background, sex, and pathogenic virulence factors that can drive microbial growth, dissemination, and tissue colonization, could result in their persistence, and also manifestations of more severe diseases. Tick-borne co-infections are increasing in North America and Europe because they can be acquired simultaneously or sequentially by the same species of infected ticks during blood meal (17, 43, 44). In the United States, tick-borne co-infections have been studied to some extent in the prominent reservoir host, white-footed mouse (*Peromyscus leucopus*), due to its higher competence in maintaining and transmitting pathogens to ticks (45–47). Understanding the interaction between *B. burgdorferi* and *B. microti* in susceptible murine infection models provides insights into their interactions in host and improves our knowledge about the pathogenic mechanisms that could be extrapolated to some level for human disease. Therefore, we examined the effects of coexisting pathogens, *B. burgdorferi* and *B. microti,* on the survival and persistence of each pathogen and respective disease outcomes. We used C3H/HeJ mice, a strain known for mutations in the TLR4 gene (28) that shows more pronounced LD symptoms (26, 33), and carried out experiments to determine the impact of both short-term (2–4 weeks) and long-term (8–16 weeks) infection on host and pathogens.

We observed that *B. burgdorferi* attenuates *B. microti* peak parasitemia in both female and male C3H/HeJ co-infected mice at the acute phase of infection, with a more pronounced difference seen in male mice (Fig. 2A). During long-term infection (16 weeks pi), parasitemia levels examined microscopically in all experimental groups infected with *B. microti*, irrespective of co-infection, were very low (<1%), with no significant differences observed between different groups (Fig. 2A, Table 1). We previously showed that *B. burgdorferi* infection significantly attenuates *B. microti* parasitemia in both C3H/HeJ and C3H/HeN mice at the early stages of infection (27, 48). Attenuation of peak parasitemia in co-infected C3H mice, when compared to mice infected solely with *B. microti,* was confirmed by another study (25). In contrast, laboratory-bred white-footed mice co-infected with another strain of *B. burgdorferi* (B348) via tick bites had significantly higher parasitemia compared to those infected solely with *B. microti* (49), suggesting differences in the outbred reservoir host and inbred C3H mice. In addition, our N40 strain of *B. burgdorferi* is highly invasive, while the B348 strain was considered non-invasive (50, 51). N40 efficiently disseminates through the bloodstream and consequently affects the immune response required to control *B. microti* (52). Thus, both the strain of infecting microbes and genotype of the host influence the interaction between pathogens and their burden. We believe that the innate immune response against the invasive N40 strain can control and attenuate the parasitemia in co-infected mice, which may not occur with the non-invasive B348 strain (53). Furthermore, the inoculum used for *B. burgdorferi* and *B. microti* iRBCs was 10^3^ and 10^5^ in another study (24), while we used 10^3^ and 10^4^ spirochetes and iRBCs, respectively. The age of mice or time points of observation also influence *B. microti* parasitemia, as demonstrated by lower parasitemia levels in older (30-week-old) compared to younger mice (25).

The interaction between *B. burgdorferi* and *B. microti* differentially affects disease outcomes. Our results at 2 weeks pi (Fig. 3) confirm our previous reports where N40 *+ B. microti* co-infected C3H mice at the acute phase (up to 3 weeks) showed higher spirochete burden compared to N40-infected mice. In the previous studies, increased bioluminescent N40 colonization in co-infected mice was also confirmed by qPCR analyses of tissue spirochete load in brains and joints (26, 27). In this study, the burden of spirochetes in joints at 4 and 16 weeks of infection was >10-fold reduced to our previous results at 3 weeks post-infection. Long-term (at 16 weeks pi) *B. microti* parasitemia remained barely detectable across all *B. microti*-infected groups. Many protozoan infections can induce immunosuppression in hosts (54–58), including *B. microti* (52, 59). Some studies have suggested that *B. microti* co-infections have immunosuppressive effects to enhance the persistence of co-infecting agents (27, 60, 61). *B. microti* infection was shown to reduce splenic B and T cell percentage resulting in diminished humoral response against both pathogens in C3H mice (27). Failure to produce specific antibodies against the extracellular pathogen *B. burgdorferi* can lead to a persistent infection and more severe tissue damage in mammalian hosts (62). Another study has shown that *B. microti* infection is associated with the production of higher levels of interleukin (IL)−10-producing B and CD4+ CD25+ FoxP3+ regulatory T cells (63). The same study also showed that B cells isolated from *B. microti*-infected mice were capable of secreting high levels of IL-10 when stimulated *ex vitro* with LPS, the principal outer membrane component of gram-negative bacteria.

Splenomegaly is one of the most pronounced babesiosis manifestations during the acute phase, reflecting the innate immune response against *B. microti* through splenic macrophages (39, 52, 64). Severe babesiosis in the splenectomized patients is associated with high morbidity and mortality, underscoring the essential function of the spleen in disease resolution (5). Pronounced splenomegaly at 2 to 3 weeks pi (24, 25, 27) was largely resolved in male mice but persisted in female mice even at 16 weeks pi (Fig. 2B). Since the spleen plays a crucial role in *Babesia* infection clearance and adaptive immunity, serving as a major site for antigen presentation, B cell activation, and antibody production (65), its prolonged enlargement in female mice may indicate a sustained immune response or delayed resolution of disease. *B. microti* parasitemia was very low across all infected groups at 16 weeks; however, *B. burgdorferi* burden remained significant in all infected mice at 8 and 16 weeks pi (Fig. 3B). We did not directly assess splenic immune cell populations or cytokine profiles in this study. Splenomegaly has been linked to individuals sero-reactive against *Babesia* and *B. burgdorferi* antigens (19); however, the relation between co-infection and babesiosis-associated splenomegaly in humans remains unclear. Fundamentally, Th1 cell-mediated immunity and marked increase in macrophage levels were associated with resolution of *B. microti* parasitemia in mice, yet despite parasite clearance, recovery of internal organs, including the spleen, may be delayed, prolonging illness (27). The severity of the late-stage *B. microti* infection outcomes may be more serious in humans due to unidentified determinants that could trigger spontaneous splenic rupture, even in asymptomatic individuals (6–12). Notably, this phenomenon has not been reported in any strain of mice susceptible to *Babesia* infection, despite their ability to develop other manifestations of babesiosis. Analysis of the systemic and splenic immune response in infected and co-infected mice could help clarify the role or inadequacy of innate and adaptive immune responses to *B. burgdorferi*.

Lyme neuroborreliosis (LNB) is one of the most common complications of LD affecting 10%–20% of patients who were also unresponsive to antibiotics (66). In part, LNB was attributed to the invasion of the central nervous system (CNS) by the Lyme spirochetes in humans (67). Other pathogens can also be neuropathogenic because they utilize several strategies to transmigrate across the BBB, such as transcellular, paracellular, and Trojan horse routes (68). An early report showed that co-infection with other tick-borne pathogens enhances the transmigration of *B. burgdorferi* across the human BBB, which could be an aggravating factor of neuroborreliosis (69). Using immunofluorescence assay, we previously showed that during co-infection with *B. microti,* more N40 spirochetes were detected in the brain at the acute phase of infection (27). However, the levels of bacteria were not high enough to produce detectable bioluminescence in dense brain tissue, making it difficult to demonstrate direct penetration of the brain by live imaging here (Fig. 5B). Given the ability of *B. burgdorferi* and *B. microti* to reach the CNS and that the co-infection plays an important role in increasing N40 persistence during long-term infection, we assessed BBB integrity in mice at the late stage of infection. Although bioluminescent detection showed that N40 colonizes the dura mater at 2 weeks pi (Fig. 5B), BBB disruption was not observed in N40-infected mice at any stage of infection (Fig. 5B and data not shown). It is not unexpected because thin spiral *B. burgdorferi* likely penetrate the meninges through intercellular spaces without disrupting the BBB. Less extravasation of Evans Blue was also reported in the brain and lungs of *P. berghei-B. microti* co-infected mice compared to the group infected with *P. berghei* alone. In addition, *B. microti* co-infection decreased *P. berghei* sequestration and ameliorated tissue injury, which culminated in attenuation of cerebral malaria severity. IFN-γ, TNF-α, and IL-12p70 levels also reduced in the sera of these mice (70).

Several studies have shown that *B. burgdorferi* invades distant organs, including the CNS, by passing through endothelial barriers mediated by different types of host matrix metalloproteases (MMPs) and pro-urokinase-type plasminogen activators, which are also involved in tissue remodeling and immune cell extravasation to enhance spirochete levels within the CNS (69, 71–75). The dura mater was shown to be colonized by B31 and 297 spirochete strains in experimentally infected C3H/HeN mice with *B. burgdorferi* presence in vascular, perivascular, or extravascular regions even at 10 weeks pi (76). The peak of spirochete burden within the dura mater was reported to occur as early as 7 days post-infection (77). We did not observe significant differences in N40 colonization of the dura mater between N40-infected and N40 *+ B. microti* co-infected mice, at 2 weeks pi (Fig. 5B); however, minimal bioluminescence in the dura mater was detected only in co-infected mice at 16 weeks pi (data not shown). Interestingly, co-infection with *Anaplasma phagocytophilum* contributes to higher *B. burgdorferi* burden, persistence, Lyme arthritis severity (78), and transmigration of spirochetes through endothelial junctions such as those found in the BBB, without affecting endothelial cell integrity (69). More studies are needed in murine models to understand the mechanisms by which *B. burgdorferi* penetrates the BBB to reach the dura mater and how the co-infection with *B. microti* can potentially contribute to spirochete persistence in humans, contributing to the severity of neuroborreliosis.

We previously showed that co-infection with *B. microti* exacerbates Lyme arthritis up to 3 weeks pi (25–27). In this study, we found that the severity of inflammatory arthritis was ameliorated at persistent stages of infection. Interestingly, although the burden of spirochetes had reduced even at 4 weeks of infection (Fig. 4), pronounced inflammatory arthritis remained at this stage, persisting until 16 weeks pi (Fig. 6, Table 2), likely because of the release of pro-inflammatory antigens from dead spirochetes, such as peptidoglycans (79–81) and other yet-to-be-discovered molecular remnants. Lyme inflammatory arthritis is a very common manifestation observed in individuals with long-term LD (2) and experimental murine models (82–84) and can worsen also during co-infection with obligate intracellular tick-transmitted bacterial pathogen, *A. phagocytophilum*, which also results in higher *B. burgdorferi* burden and persistence (78), as observed during co-infections with *B. microti* at the acute phase (25–27). The Th1-like immune response plays an important role in proinflammatory outcomes of LD, as evidenced by its prevalence in the synovial fluid from long-term LD patients suffering from inflammatory arthritis. A higher ratio of Th1 to Th2 cells in the synovial fluid correlates with the increased severity of Lyme arthritis. In contrast, Th2 immunity is implicated in inflammation resolution and autoimmune processes (85). C3H/HeJ mice are an ideal model for the study of Lyme arthritis because they also develop an IFN-γ-producing Th1 response and expansion of CD4+ T cells subsets that are heavily involved in the development of severe arthritis (86, 87). The interleukin-4 role is protective against Lyme arthritis in BALB/c and C3H mice due to its involvement in downregulation of the Th1 response and in the development of humoral immunity against spirochetes (88). Based on the literature and our findings (26), we believe that CD4+ Th1 cells have an important role involved in the clearance of N40 and resolution of Lyme arthritis, even during long-term infection.

To summarize, our results suggest that both N40 and *B. microti* survive in mice at detectable levels until 16 weeks pi. Furthermore, peak *B. microti* parasitemia was significantly lower in co-infected male mice; however, parasitemia reduced significantly in both male and female mice at 4 weeks of infection, while significant splenomegaly remained only in female mice at 16 weeks pi. Lyme arthritis persisted in both N40-infected and co-infected mice at this late stage of infection. Persistence of Lyme spirochetes and associated tissue inflammation during late infection require further investigation in humans, irrespective of co-infections. Given that the etiology of long-term and severe Lyme disease symptoms remains poorly understood in most LD patients, future studies including on co-infections would help clarify the mechanisms involved and their impact on patients’ quality of life.

## SUPPLEMENTAL MATERIAL

Figure S1 spectrum.00252-25-s0001.docx Measurement of bioluminescence radiance depicted in Figure 2 in male and female mice infected with N40 alone or with *Babesia microti*.

ASM does not own the copyrights to Supplemental Material that may be linked to, or accessed through, an article. The authors have granted ASM a non-exclusive, world-wide license to publish the Supplemental Material files. Please contact the corresponding author directly for reuse.
