# Supplementary material for: Long-term survival of Babesia microti and Borrelia burgdorferi in C3H/HeJ mice and their effect on Lyme arthritis and babesiosis manifestations
Source: Microbiol Spectr. 2025 Aug 12;13(9):e00252-25. doi: 10.1128/spectrum.00252-25 (PMC12403850; doi:10.1128/spectrum.00252-25)
Supplement: Figure S1 — Measurement of bioluminescence radiance depicted in Figure 2 in male and female mice infected with N40 alone or with Babesia microti. [file spectrum.00252-25-s0001.docx]

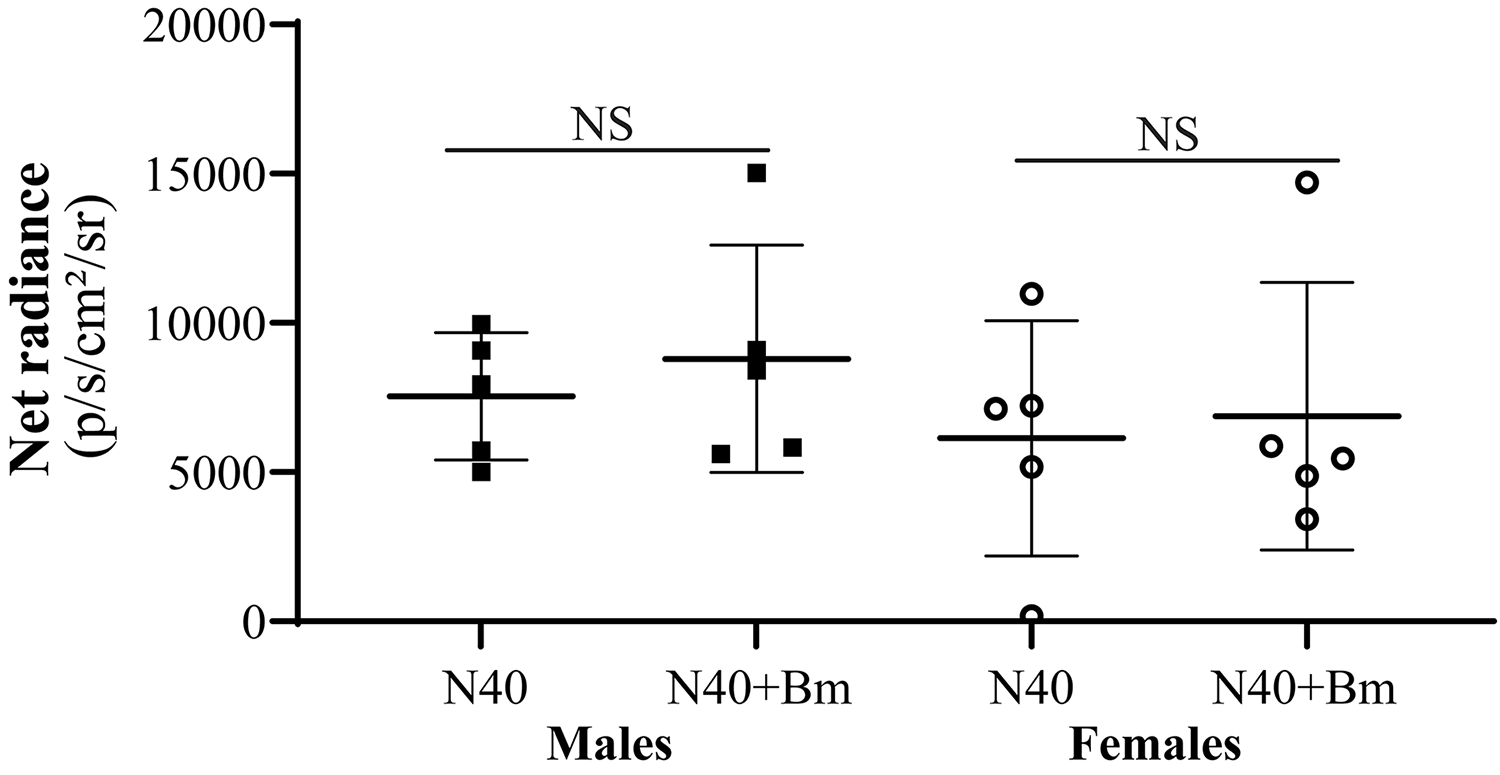


Figure S1. Measurement of bioluminescence radiance depicted in Figure 2 in male and female mice infected with N40 alone or with B. microti.
